# Synthetic engineering of *Corynebacterium crenatum* to selectively produce acetoin or 2,3-butanediol by one step bioconversion method

**DOI:** 10.1186/s12934-019-1183-0

**Published:** 2019-08-06

**Authors:** Xian Zhang, Rumeng Han, Teng Bao, Xiaojing Zhao, Xiangfei Li, Manchi Zhu, Taowei Yang, Meijuan Xu, Minglong Shao, Youxi Zhao, Zhiming Rao

**Affiliations:** 1 The Key Laboratory of Industrial Biotechnology, Ministry of Education, School of Biotechnology, Jiangnan University, 1800 Lihu Road, Wuxi, 214122 Jiangsu China; 2 Department of Chemical and Biomolecular Engineering, The Ohio State University, Columbus, OH 43210 USA; 3 School of Life Science and Technology, ShanghaiTech University, 393 Middle Huaxia Road, Shanghai, 201210 China; 4 Beijing Key Laboratory of Biomass Waste Resource Utilization, College of Biochemical Engineering, Beijing Union University, Beijing, 10023 People’s Republic of China

**Keywords:** *Corynebacterium crenatum*, Synthetic engineering, Biocatalysis, Acetoin, 2,3-Butanediol

## Abstract

**Background:**

Acetoin (AC) and 2,3-butanediol (2,3-BD) as highly promising bio-based platform chemicals have received more attentions due to their wide range of applications. However, the non-efficient substrate conversion and mutually transition between AC and 2,3-BD in their natural producing strains not only led to a low selectivity but also increase the difficulty of downstream purification. Therefore, synthetic engineering of more suitable strains should be a reliable strategy to selectively produce AC and 2,3-BD, respectively.

**Results:**

In this study, the respective AC (*alsS* and *alsD*) and 2,3-BD biosynthesis pathway genes (*alsS*, *alsD*, and *bdhA*) derived from *Bacillus subtilis* 168 were successfully expressed in non-natural AC and 2,3-BD producing *Corynebacterium crenatum*, and generated recombinant strains, *C. crenatum* SD and *C. crenatum* SDA, were proved to produce 9.86 g L^−1^ of AC and 17.08 g L^−1^ of 2,3-BD, respectively. To further increase AC and 2,3-BD selectivity, the AC reducing gene (*butA*) and lactic acid dehydrogenase gene (*ldh*) in *C. crenatum* were then deleted. Finally, *C. crenatum*Δ*butA*Δ*ldh* SD produced 76.93 g L^−1^ AC in one-step biocatalysis with the yield of 0.67 mol mol^−1^. Meanwhile, after eliminating the lactic acid production and enhancing 2,3-butanediol dehydrogenase activity, *C. crenatum*Δ*ldh* SDA synthesized 88.83 g L^−1^ of 2,3-BD with the yield of 0.80 mol mol^−1^.

**Conclusions:**

The synthetically engineered *C. crenatum*Δ*butA*Δ*ldh* SD and *C. crenatum*Δ*ldh* SDA in this study were proved as an efficient microbial cell factory for selective AC and 2,3-BD production. Based on the insights from this study, further synthetic engineering of *C. crenatum* for AC and 2,3-BD production is suggested.

**Electronic supplementary material:**

The online version of this article (10.1186/s12934-019-1183-0) contains supplementary material, which is available to authorized users.

## Background

As important platform compounds, acetoin (AC) and 2,3-butanediol (2,3-BD) are widely used in food, medicine and chemical industries [1–4]. In addition, AC and 2,3-BD production by microbial fermentation and biotransformation have drawn attentions since the environment crisis [5].

In recent years, consolidate efforts have been made towards increasing AC and 2,3-BD production by inactivation of competitive pathways or overexpression of key genes of AC and 2,3-BD biosynthesis pathways [6]. Nielsen et al. strengthened the expression of *α*-acetolactate synthase (ALS), *α*-acetolactate decarboxylase (ALDC) and 2,3-butanediol dehydrogenase (BDH) to increase the titer of AC (0.87 g L^−1^) and 2,3-BD (1.12 g L^−1^) in *Escherichia coli* [7]. The similar strategy of overexpressing ALS and ALDC was also applied to *Bacillus subtilis* and *Saccharomyces cerevisiae*, in which the productivity of AC from glucose was increased by approximately 62.9% and 58%, respectively [8, 9]. By further expressing the homogenous BDH1 in engineered *S. cerevisiae*, 2,3-BD was further increased to 96.2 g L^−1^ [10]. On the other hand, Ji et al. increased the yield of 2,3-BD to 0.48 g g^−1^ by reducing the biosynthesis of lactic acid and acetic acid in *Klebsiella oxytoca* [11]. Wang et al. knocked out *bdhA*, *pta* and *acoA* in *B. subtilis* and increased the yield of AC to 0.49 g g^−1^ [12]. More recently, Erian et al. increased the 2,3-BD titer about 2.4-fold by eliminating the lactate formation in engineered *E. coli* [13]. Meanwhile, strains of non-natural AC and 2,3-BD producing strains were metabolically engineered by expressing AC and 2,3-BD biosynthesis pathways derived from *Serratia marcescens*, *Klebsiella pneumoniae*, *Enterobacter* and *S. cerevisiae* [3, 5, 14]. Took the advantages of photosynthesis by converting light energy to chemical energy, Oliver et al. introduced a novel synthetic pathway into *Cyanobacteria*, and successfully produced AC and 2,3-BD using CO_2_ [15]. In addition, *Corynebacterium glutamicum* was also engineered for 2,3-BD production by expressing gene cluster of 2,3-BD biosynthetic pathway from *Lactococcus lactis* [16] or the *budABC* cluster from *K. pneumoniae* [17], which synthesized 6.3 and 18.9 g L^−1^ of 2,3-BD, respectively. After multistage modification, *C. glutamicum* could also be used to produce optically pure d-(−)-AC [18]. However, these AC and 2,3-BD producing strains also have some drawbacks because by-products, such as lactic acid and acetic acid, and mutually transition between AC and 2,3-BD not only lead to a low yield but also increase the difficulty of products purification.

Compared to AC and 2,3-BD fermentation, AC and 2,3-BD biocatalysis generally present a higher yield and selectivity [19, 20]. Efficiently mutual transformation of AC and 2,3-BD were realized by regenerating the intracellular NAD^+^/NADH levels in *E. coli* and *B. subtilis* [21, 22]. However, AC and 2,3-BD as substrates are both expensive and thus are not feasible for commercialization strategies by biocatalysis. Thus, to better meet industrial production needs, we urged to find more suitable host strains and more efficient biocatalyst processes. In this study, we aimed to develop one-step bioconversion from glucose to selective AC and 2,3-BD production, and chose to engineer non-natural AC and 2,3-BD producing *Corynebacterium crenatum* SYPA5-5 as the host since the following reasons: *C. crenatum*, genetically close to *C. glutamicum*, has the advantages of growing fast, non-sporulation, and easy gene editing [23]. In addition, the BDH identified in *C. crenatum* was demonstrated that catalyze both AC and diacetyl (DA) for 2,3-BD formation [24]. Therefore, synthetic engineering *C. crenatum* SYPA5-5 should be a reliable strategy to selectively produce AC and 2,3-BD, respectively.

**Fig. 1:**
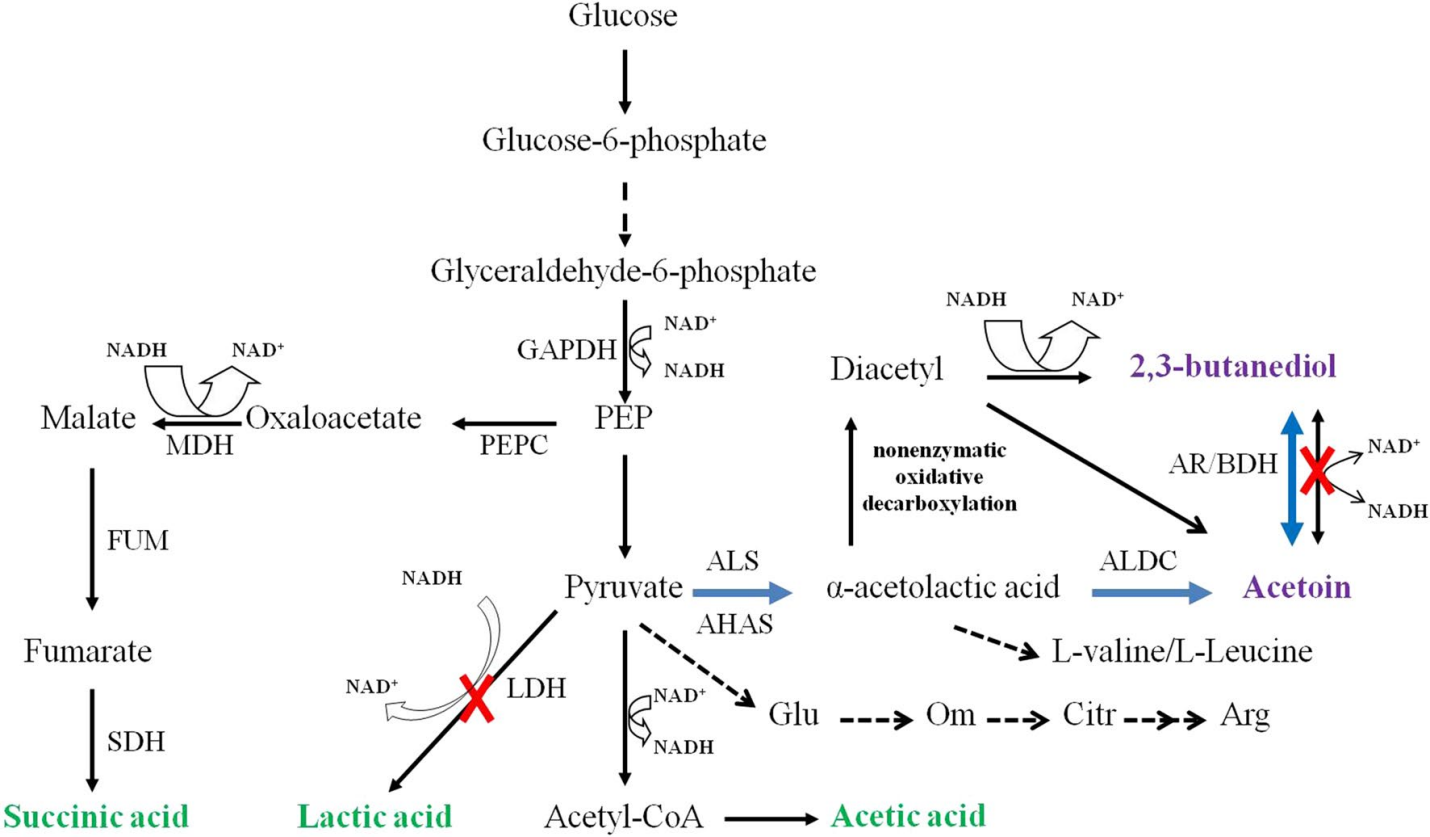
AC and 2,3-BD pathway construction in *C. crenatum*. The *blue arrow* indicate AC and 2,3-BD synthetic pathways from *B. subtilis* 168. The *red cross marks* indicate the disrupted pathways. GAPDH (glyceraldehyde-3-phosphate dehydrogenase), PEPC (phosphoenolpyruvate carboxylase), MDH (malic dehydrogenase), FUM (fumarase), SDH (succinic dehydrogenase)

In this work, AC (*alsS* and *alsD*) and 2,3-BD biosynthesis pathway genes (*alsS*, *alsD*, and *bdhA*) from *B. subtilis* 168 were overexpressed in *C. crenatum*Δ*ldh* and *C. crenatum*Δ*butA*Δ*ldh*, respectively, in which glucose was specifically transformed into AC or 2,3-BD in one-step biocatalysis (Fig. 1). This work provides a simple and efficient bioconversion process to selectively convert glucose to high value-added chemicals of AC and 2,3-BD by engineered *C. crenatum* as microbial cell factory.

## Results and discussion

### Overexpression of ALS, ALDC, and AR/BDH in *C. crenatum*

Here, an artificial operon including *alsS*, *alsD*, and *bdhA* from *B. subtilis* was selected to overexpress in *C. crenatum* to investigate its effect on AC and 2,3-BD production. The confirmed recombinants were named *C. crenatum*/pXMJ19-*alsS* (*C. crenatum* S), *C. crenatum*/pXMJ19-*alsD* (*C. crenatum* D), *C. crenatum*/pXMJ19-*bdhA* (*C. crenatum* A), *C. crenatum*/pXMJ19-*alsSD* (*C. crenatum* SD), and *C. crenatum*/pXMJ19-*alsSD*-*bdhA* (*C. crenatum* SDA), respectively.

The SDS-PAGE was used to confirm the expression of ALS (AHAS), ALDC, and AR/BDH in *C. crenatum* recombinants (Additional file 1: Figure S1). As expected, ALS (AHAS), ALDC, and AR/BDH on gel demonstrated their respective molecular mass about 60, 29, and 38 kDa, respectively. Enzyme activity assays also confirmed the functional expression of ALS (AHAS), ALDC, and AR/BDH in *C. crenatum* transformants. As shown in Table 1, the wild type strain showed negligible acetohydroxyacid synthase (AHAS) activity, which might be inhibited by the anabolism of l-valine and l-leucine in *C. crenatum* [25, 26]. Meanwhile, no ALDC activity was detected in *C. crenatum* WT. After enhanced expression of ALS and ALDC, the enzyme activities in *C. crenatum* SD and *C. crenatum* SDA was increased to 2.22/2.32 and 8.08/5.68 U mg^−1^, respectively. However, it should be noted that the lower increased activities than that of only ALS or ALDC expression probably due to lower efficiency in the tandem co-expression driven by one promoter for two or three genes. On the other hand, compared to *C. crenatum* WT, *C. crenatum* SDA showed a high AR and BDH activities (0.32 and 0.10 U mg^−1^) comparable to *C. crenatum* A (0.38 and 0.19 U mg^−1^). In addition, although AR/BDH was a bidirectional reversible enzyme [24, 27], the heterogenous AR/BDH from *B. subtilis* present a higher AR activity than BDH activity in *C. crenatum* A and *C. crenatum* SDA. With ALS, ALDC, and (or) AR/BDH, the recombinant *C. crenatum* can catalyze the pyruvate to either AC or DA, thus allowing the respective conversion of glucose to AC and 2,3-BD.

**Table 1:** The specific enzyme activities of ALS (AHAS), ALDC, and AR/BDH were determined in recombinant *C. crenatum* cell extracts

Strains	Specific enzyme activity (U mg^−1^)		
	ALS (AHAS)	ALDC	AR/BDH
*C. crenatum* WT	0.01 ± 0.01	0	0.05 ± 0.01/0.01 ± 0.01
*C. crenatum* S	4.86 ± 0.85	nd	nd
*C. crenatum* D	nd	13.59 ± 1.22	nd
*C. crenatum* A	nd	nd	0.38 ± 0.03/0.19 ± 0.01
*C. crenatum* SD	2.22 ± 0.43	8.08 ± 0.94	nd
*C. crenatum* SDA	2.32 ± 0.31	5.68 ± 0.87	0.32 ± 0.02/0.10 ± 0.01

Using *C. crenatum* WT as positive control, 1% recombinant strains were transferred to 10 mL LBG medium with 10 g mL^−1^ chloramphenicol. After 12 h of activation, 1% bacterial solution was transferred to 50 mL LBG liquid medium. After incubation for 3–4 h at 30 °C, 180 r min^−1^, the expression of heterologous enzyme in the recombinants was induced by adding 1 mM IPTG and incubated for 12 h. Specific enzymatic activities of ALS (AHAS), ALDC, and AR/BDH in the crude enzyme solution were assayed after disrupting the recombinant cells with sonicator. The specific enzyme activity results are the mean ± standard of three replicates

*nd* not detected

### Production of AC and 2,3-BD by shake flask fermentation of recombinant *C. crenatum*

Batch fermentations with 120 g L^−1^ glucose as the sole carbon source were first performed in shack flask to investigate the metabolic impact of ALS, ALDC, and BDH expression. Since *C. crenatum* is a facultative anaerobe, cell growth under different dissolved oxygen condition often leads to generate different products [28, 29]. Previous study has demonstrated that *C. crenatum* shows a fast growth rate of biomass when using glucose as the substrate, and is diffusely used in the yielding of sundry amino acids such as l-glutamic acid and l-arginine under sufficient oxygen supply conditions [28]. Therefore, the by-product l-arginine was also determined at the end of fermentation in this study. The results are shown in Table 2.

**Table 2:** Fermentation kinetics by recombinant *C. crenatum* in shack flask

Strain	Time (h)	Glucose consumption (g L^−1^)	Glucose uptake rate (g L^−1^ h^−1^)	Diacetyl (g L^−1^)	Acetoin (g L^−1^)	2,3-Butanediol (g L^−1^)	l-Arginine (g L^−1^)
*C. crenatum* WT	120	110 ± 2	0.91 ± 0.02	nd	nd	nd	23.59 ± 1.7
*C. crenatum* S	120	36 ± 4	0.30 ± 0.03	2.17 ± 0.13	0.76 ± 0.12	0.45 ± 0.13	7.09 ± 0.70
*C. crenatum* D	120	78 ± 3	0.65 ± 0.02	nd	nd	nd	15.66 ± 1.31
*C. crenatum* A	120	73 ± 4	0.61 ± 0.03	nd	nd	nd	14.27 ± 1.45
*C. crenatum* SD	120	70 ± 3	0.58 ± 0.02	nd	13.59 ± 1.25	10.61 ± 1.33	8.74 ± 1.12
*C. crenatum* SDA	120	70 ± 6	0.58 ± 0.05	nd	9.43 ± 1.50	15.16 ± 1.31	7.83 ± 0.84

Using *C. crenatum* WT as positive control, 10% recombinant *C. crenatum* was transferred to the fermentation medium containing 120 g L^−1^ glucose for shake flask fermentation (120 g L^−1^ glucose was used as substrate). The titer of DA, AC, 2,3-BD and l-arginine were determined after 120 h of fermentation. The results of the fermentation parameters are the mean ± standard of three biological replicates

*nd* not detected

As expected, *C. crenatum* WT yield 23.6 g L^−1^ l-arginine, and no AC and 2,3-BD were detected in the fermentation broth. Theoretically, overexpression of *alsS*, *alsD*, and *bdhA* gene, respectively, cannot produce AC and 2,3-BD in *C. crenatum*. However, except for little AC and 2,3-BD producing, *C. crenatum* S also produced 2.17 g L^−1^ of unexpected DA, which could not be accumulated in *B. subtilis* [30]. This might be due to the spontaneous non-enzymatic decarboxylation reaction of *α*-acetolactate under natural oxidative aerobic conditions [31]. In addition, our previous work has also proved that the BDH from *C. crenatum* could catalyze DA to AC and then convert to 2,3-BD [24]. This explains why the chain reaction was got through in *C. crenatum* S without expression of ALDC. Moreover, it has been reported that DA is a bacteriostatic food additive that can inhibit bacteria growth [3]. Thus, *C. crenatum* S consumed only about 36 g L^−1^ glucose and then suspended the fermentation while accumulated comparable amount of DA, leading to a low efficiency of the multiple-step reaction from pyruvate to AC and 2,3-BD.

On the contrary, after introducing the *alsSD* operon with one promoter into *C. crenatum*, co-expression of ALS and ALDC effectively converted pyruvate to 13.59 g L^−1^ AC and 10.61 g L^−1^ 2,3-BD, respectively. To enhance 2,3-BD production, *bdhA* was further expressed in *C. crenatum* SD and the generated *C. crenatum* SDA dramatically increased 2,3-BD titer by 43%. These results indicated that ALS and ALDC are both important for AC and 2,3-BD biosynthesis in *C. crenatum*. In addition, enhanced heterogeneous BDH activity was confirmed the positive effect on 2,3-BD biosynthesis. However, *C. crenatum* SD and *C. crenatum* SDA still produced comparable amount of l-arginine as by-product, resulting in a low yield of AC and 2,3-BD. Therefore, l-arginine biosynthesis must be decreased.

### Bioconversion of glucose to AC and 2,3-BD by recombinants *C. crenatum* resting cells

l-Arginine as growth factor is necessary for *C. crenatum* growth, which cannot be directly depressed by gene knock-out. l-arginine production by *C. crenatum* needs high level of dissolved oxygen, while there is no strict demand on dissolved oxygen for the acid-butanediol fermentation. Therefore, we attempted to produce AC and 2,3-BD using resting cell bioconversion with glucose as substrate by *C. crenatum* SD and *C. crenatum* SDA, respectively.

**Fig. 2:**
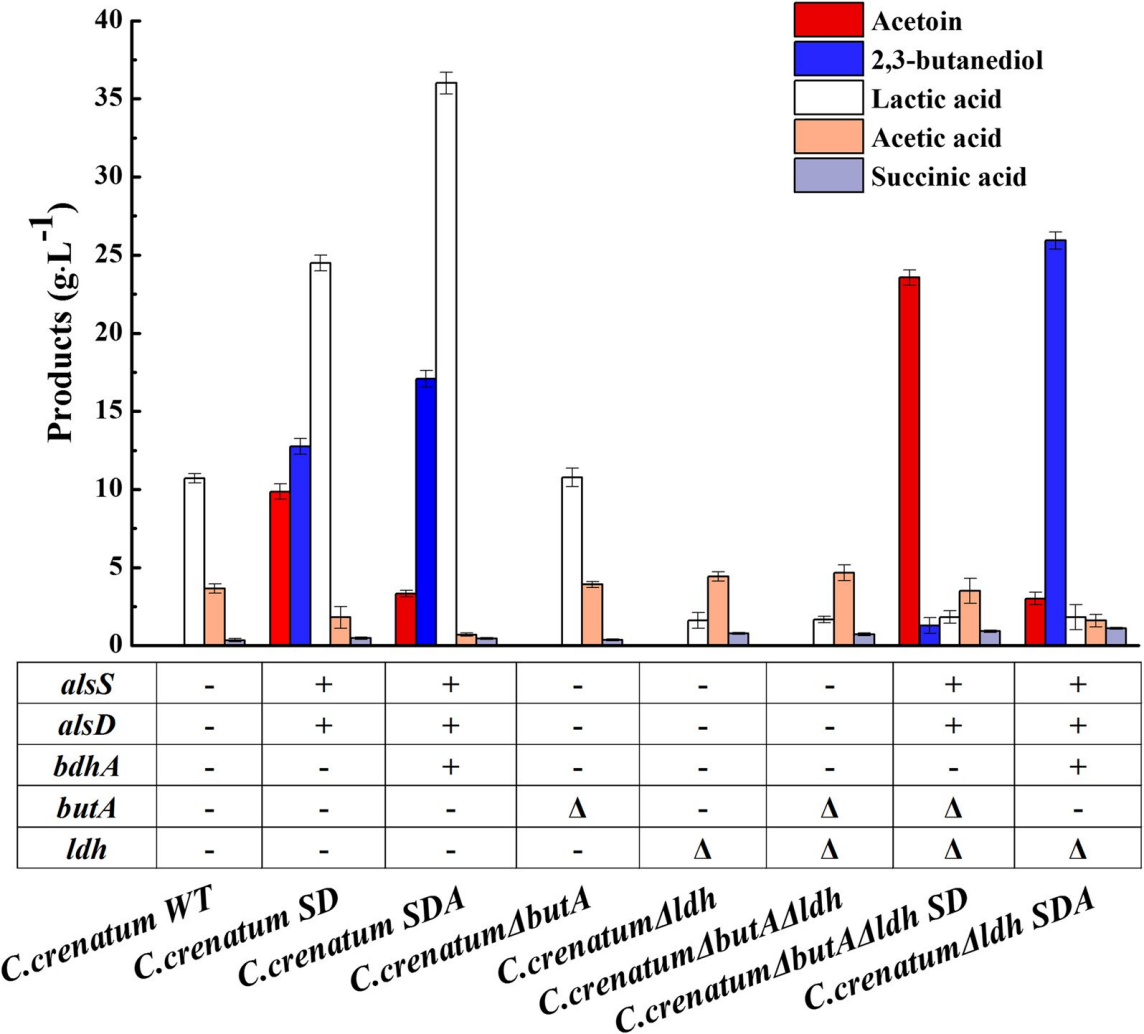
Resting cell bioconversion analysis of *C. crenatum* and the recombinant strains in shack flask. Recombinant *C. crenatum* (100 µL) was transferred to 10 mL LBG medium and incubated for approximately 12 h at 30 °C, 180 r min^−1^. The cultured bacterial solution (3 mL) was transferred into 30 mL of fermentation medium at the same culture condition for 24 h. The cultured cells were harvested by centrifugation at 8000 r min^−1^ for 10 min and then resuspended in resting cell bioconversion medium (containing 100 g L^−1^ glucose) for resting cell bioconversion. Formed metabolites and organic acid during the bioconversion of resting cells are shown. The results of resting cell bioconversion kinetics are shown as the mean ± standard of three replicates. +: overexpression, Δ: knockout

As demonstrated in Fig. 2, no l-arginine was detected during the resting cell bioconversion process (data not shown). *C. crenatum* SD converted 100 g L^−1^ glucose to 9.86 g L^−1^ AC and 12.76 g L^−1^ 2,3-BD, respectively. Similarly, *C. crenatum* SDA further accumulated 2,3-BD to 17.08 g L^−1^ with a high 2,3-BD/AC ratio. However, the content of organic acids, especially lactic acid, were also detected in both *C. crenatum* SD and *C. crenatum* SDA, which was consistent with previous study that *C. crenatum* can produce various organic acids such as succinic acid, acetic acid and lactic acid in the case of limited dissolved oxygen [29]. As the accumulation of organic acids increases the consumption of carbon sources [32, 33], the synthetic pathways of organic acids may competitively inhibit the accumulation of AC and 2,3-BD.

### Construction of *ldh* and *butA* blocked recombinant *C. crenatum*

Although resting cell bioconversion reduced l-arginine formation, some pyruvate was still converted to lactic acid. In addition, unexpected high yield of 2,3-BD was still produced by *C. crenatum* SD. To further decrease by-products and enhance the AC and 2,3-BD selectivity, blocking the competitive synthesis pathways in *C. crenatum* was considered to be necessity. The homologous lactate dehydrogenase (*ldh*) and butanol dehydrogenase (*butA*) genes in *C. crenatum* were knocked out to increase respective AC and 2,3-BD selectivity. The suicide vectors pK18-Δ*ldh* (Additional file 2: Figure S2) and pK18-Δ*butA* (Additional file 3: Figure S3), respectively, was constructed and transformed into *C. crenatum*. The mutant strains *C. crenatum*Δ*ldh* and *C. crenatum*Δ*butA* were finally obtained after two rounds of homologous recombination. Then, pK18-Δ*ldh* was further introduced into *C. crenatum*Δ*butA* and generated *C. crenatum*Δ*butA*Δ*ldh*. All of mutant strains were verified by PCR using the upstream and downstream primers of *ldh* and *butA* (Additional file 4: Figure S4).

LDH and AR/BDH activities of *C. crenatum* mutants were determined, and the results were shown in Table 3. LDH activity was reduced by 83–88% after blocking of *ldh* gene. Meanwhile, AR and BDH activities were both reduced by 62–82% and 83–87%, while *butA* gene was knocked out. The results indicated that the AC reduction and lactic acid biosynthesis pathway were depressed by knocking out *butA* and *ldh* genes. By comparing the dry cell weights of *C. crenatum* WT and the knockout strains (Additional file 5: Table S1), it was found that the knockout of *ldh* and *butA* genes had little effect on the growth of the bacteria.

**Table 3:** Specific enzyme activity assays of LDH and AR/BDH in crude cell extracts of recombinant *C. crenatum* strains

Strains	LDH (U mg^−1^)	AR/BDH (U mg^−1^)
*C. crenatum* WT	1.04 ± 0.03	0.055 ± 0.003/0.003 ± 0.001
*C. crenatum*Δ*ldh*	0.18 ± 0.01	nd
*C. crenatum*Δ*butA*	nd	0.021 ± 0.003/0.0005 ± 0.001
*C. crenatum*Δ*butA*Δ*ldh*	0.13 ± 0.01	0.010 ± 0.002/0.0004 ± 0.001

Using *C. crenatum* WT as positive control, 1% of recombinant *C. crenatum* was transferred to 50 mL LBG medium and incubated in shake flask at 180 r min^−1^, 30 °C for 12 h. The supernatant of the disrupted cells was taken to LDH and AR/BDH activity assay. The results of the specific enzyme activity assays are the mean ± standard of three replicates

*nd* not detected

Then, the recombinant *C. crenatum*Δ*butA*, *C. crenatum*Δ*ldh*, and *C. crenatum*Δ*butA*Δ*ldh* were subjected to resting cell and the bioconversion results were shown in Fig. 2. As expected, after knocking out *ldh* gene, the acetic and succinic acid production were slightly increased, while the lactic acid production dramatically decreased by about 85% in *C. crenatum*Δ*ldh* and *C. crenatum*Δ*butA*Δ*ldh*. Moreover, compared with *C. crenatum* WT and *C. crenatum*Δ*ldh*, there is no significant effect on biocatalysis after *butA* delection.

### Efficient one-step bioconversion of glucose to AC by recombinant *C. crenatum*Δ*butA*Δ*ldh*/pXMJ19-*alsSD* resting cell

To construct the one-step bioconversion of glucose to AC, plasmid pXMJ19-*alsSD* was then transformed into *ldh* and *butA* deletion strain to construct *C. crenatum*Δ*butA*Δ*ldh*/pXMJ19-*alsSD* (*C. crenatum*Δ*butA*Δ*ldh* SD). *C. crenatum*Δ*butA*Δ*ldh* SD consumed 94 g L^−1^ glucose after 60 h with an uptake rate of 1.57 g L^−1^ h^−1^, which was significantly higher (2.18-fold and 2.34-fold, respectively) than that of *C. crenatum* WT and *C. crenatum*Δ*butA*Δ*ldh*, indicating that the expression of heterologous AC biosynthesis pathway strongly promoted glucose for AC production. Consequently, AC production dramatically increased from 9.86 to 23.56 g L^−1^, about 2.39-fold than before. Meanwhile, only a small amount of 2,3-BD (1.28 g L^−1^) and lactic acid (1.83 g L^−1^) were detected, resulting in an AC molar yield of up to 0.51 mol mol^−1^. Consistence with that of *C. crenatum*Δ*butA*Δ*ldh*, acetic acid and succinic acid has hardly changed (Fig. 2).

### Efficient one-step bioconversion of glucose to 2,3-BD by recombinant *C. crenatum*Δ*ldh*/pXMJ19-*alsSD*-*bdhA* resting cell

To further extend the products chain for 2,3-BD production, pXMJ19-*alsSD*-*bdhA* was introduced into *C. crenatum*Δ*ldh*, resulting in *C. crenatum*Δ*ldh*/pXMJ19-*alsSD*-*bdhA* (*C. crenatum*Δ*ldh* SDA) which co-expresses both homologous and heterologous AR/BDH. Generally, compared with *C. crenatum* SDA as the positive control, *C. crenatum*Δ*ldh* SDA resting cell converted more glucose to 2,3-BD. After 60 h, *C. crenatum*Δ*ldh* SDA consumed 95 g L^−1^ glucose with an uptake rate of 1.58 g L^−1^ h^−1^. With overexpression of AR/BDH and depressing LDH, 2,3-BD production increased from 17.08 to 25.93 g L^−1^, about 52% higher than the control. Meanwhile, lactic acid was decreased about 95%, leading to a high 2,3-BD molar yield of 0.55 mol mol^−1^ (Fig. 2). It should be noted that the bioconversion for 2,3-BD production was still accompanied by certain AC accumulation because of the reversible reaction of AR/BDH. Moreover, 2,3-BD and lactic acid are both NADH-dependent products. Therefore, depressing LDH also releases additional reducing equivalent, which further releases the constraint of co-enzyme poll on 2,3-BD synthesis. However, the yields of acetic acid (1.6 g L^−1^) and succinic acid (1.1 g L^−1^) were slightly higher than *C. crenatum* SDA.

### Repeated batch resting cell bioconversion for AC and 2,3-BD production in 5 L bioreactor

**Fig. 3:**
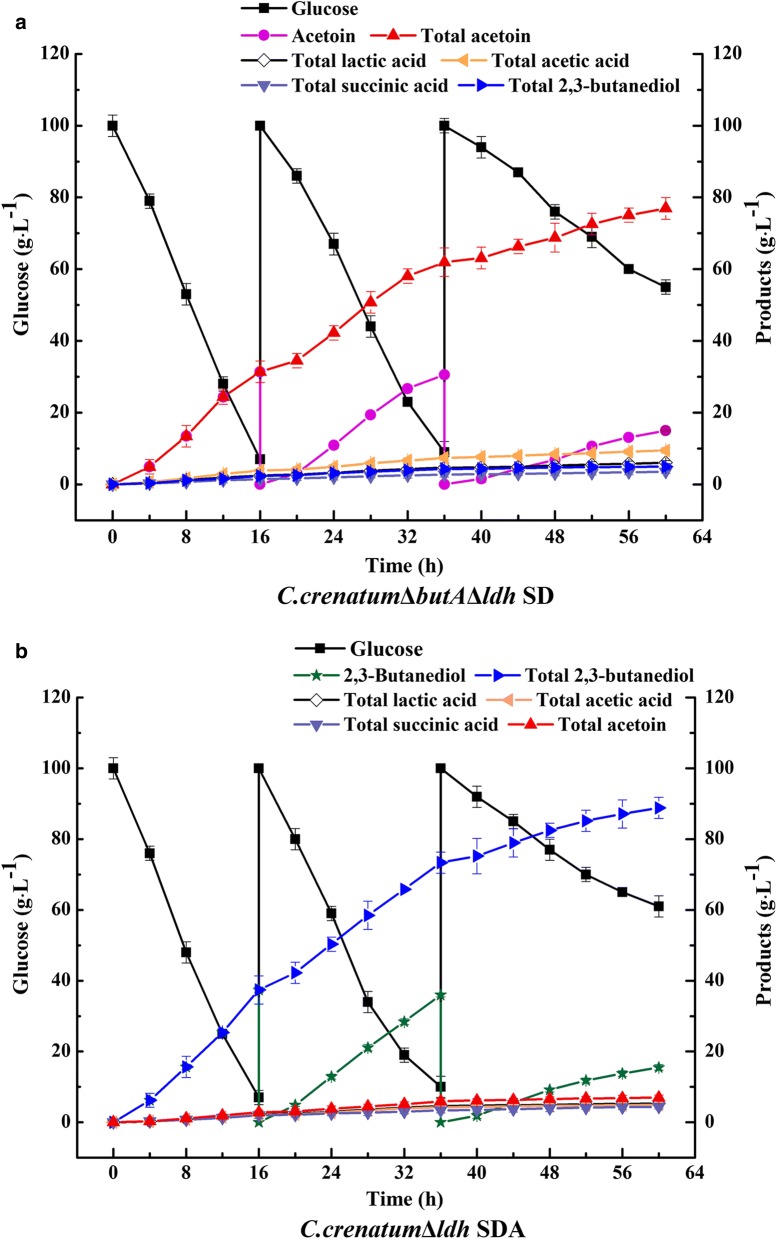
Resting cell bioconversion analysis of recombinant *C. crenatum* in 5 L bioreactor. Recombinant *C. crenatum* (200 µL) was transferred to 20 mL LBG medium and incubated for 24 h at 180 r min^−1^, 30 °C. After the 10% cultures were transferred and cultured in 200 mL seed medium for 18 h, the whole cultures were transferred to 5 L bioreactor containing 2 L of fermentation medium, and incubated for 24 h at 30 °C, 600 r min^−1^. 4 L recombinant *C. crenatum* cultures were collected and resuspended in 2 L resting cell bioconversion medium containing 100 g L^−1^ glucose. Resting cell bioconversion was performed in batches at 30 °C, 250 r min^−1^. Consumed glucose, formed metabolites, and organic acid during the bioconversion of resting cells are shown. The results of resting cell bioconversion kinetics are shown as the mean ± standard of three replicates

To investigate the long-term stability and performance of recombinant *C. crenatum*, repeated batch resting cell bioconversion were performed in 5 L bioreactors, and the results are shown in Fig. 3. Generally, the resting cell bioconversion was repeated for 3 cycles with 100 g L^−1^ of glucose as the substrate. In the first two batch conversions, AC production was stably increased by *C. crenatum*Δ*butA*Δ*ldh* SD. However, AC productivity of the third batch has gradually declined. Finally, after total 60 h of bioconversion, 76.93 g L^−1^ of AC with the yield of 0.67 mol mol^−1^ was produced by *C. crenatum*Δ*butA*Δ*ldh* SD (Fig. 3a). Meanwhile, after eliminating the lactic acid production and enhancing 2,3-butanediol dehydrogenase activity, *C. crenatum*Δ*ldh* SDA also synthesized 88.83 g L^−1^ of 2,3-BD with the yield of 0.80 mol mol^−1^ (Fig. 3b). It should be noted that both average AC and 2,3-BD productivity throughout the repeated batch conversion process reached 1.28–1.48 g L^−1^ h^−1^, which increased ~ 3.5-fold compared to flask batch biocatalysis. However, significant decrease of enzyme activities was observed during the third process (data not shown). Therefore, further strategies should be developed to maintain the cell viability and stability during the whole bioconversion process.

### Comparison with other study

Currently, microbial fermentation is still the main method for producing AC and 2,3-BD from various substrates. However, its relatively long fermentation period and low productivity and substrate conversion rate are currently obstructed the industrial application [18, 34, 35]. Biocatalysis, as a highly efficient and environmentally friendly methods, can significantly improve substrate utilization rate and product yield. As shown in Table 4, previous studies have demonstrated that engineered *E. coli* and *B. subtilis* can be used as host for biocatalytic synthesis of AC and 2,3-BD, in which the highest yield of AC and 2,3-BD can be reached to 0.98 mol mol^−1^ and 0.96 mol mol^−1^. However, most of these biocatalysts cases were carried out using AC or 2,3-BD as a substrate, which are not suitable for industrial production at all. Although 2,3-BD and AC bioconversion from glucose were achieved by *K. pneumoniae* and *B. subtilis*, two step batch strategy still cannot selectively separate mixed (2*S*,3*S*)-2,3-BD and (3*S*)-AC [41]. In this study, we developed a new mono-bioconversion system using *C. crenatum* as the only host for AC and 2,3-BD production directly from glucose. After depressing competition pathways and overexpressing AC and 2,3-BD biosynthesis genes from *B. subtilis*, one-step biosynthesis of AC and 2,3-BD, respectively, were achieved in *C. crenatum*Δ*butA*Δ*ldh* SD and *C. crenatum*Δ*ldh* SDA without additional complex nutrients.

**Table 4:** Comparison of titer and yield of AC and 2,3-BD produced by different strains through bioconversion in recent years

Strains	Bioconversion method	Substrates	Products	Titer (g L^−1^)	Yield (mol mol^−1^)	References
*E. coli*	Fed batch	DA	AC	39.4	0.83	[36]
*E. coli* BL21 (DE3)	Batch	*meso*-2,3-BD	(3*R*)-AC	86.7	0.94	[37]
*E. coli* BL21 (DE3)	Batch	*meso*-2,3-BD	(3*S*)-AC	36.7	0.88	[21]
*E. coli* BL21 (DE3)	Batch	(2*R*,3*R*)-2,3-BD	(3*R*)-AC	41.8	0.98	[21]
*B. subtilis* 168	Fed batch	2,3-BD	AC	91.8	0.78	[22]
*E. coli*	Batch	*meso*-2,3-BD	(3*S*)-AC	72.38	0.92	[38]
*E. coli*	Batch	*meso*-2,3-BD	(2*S*,3*S*)-2,3-BD	38.41	0.96	[38]
*E. coli* BL21 (DE3)	Fed batch	DA	(2*S*,3*S*)-2,3-BD	31.7	0.86	[39]
*B. subtilis* 168	Repeated batches	AC + formate	2,3-BD	115.4	0.96	[40]
*B. subtilis* 168	Batch	AC + glucose	2,3-BD	63.7	0.96	[40]
*K. pneumoniae* and *B. subtilis* 168	Two-step sequencing batch	Glucose	(3*S*)-AC	56.7	0.85	[41]
*C. crenatum*Δ*butA*Δ*ldh* SD	Repeated batches	Glucose	AC	76.9	0.67	This study
*C. crenatum*Δ*ldh* SDA	Repeated batches	Glucose	2,3-BD	88.8	0.80	This study

Compared to usual 100–120 g L^−1^ AC and 2,3-BD produced by microbial fermentation, further improvements of *C. crenatum* are necessary for industrial application. Although depressing *ldh* and *butA* and enhancing AC or 2,3-BD biosynthesis pathway activity, *C. crenatum*Δ*butA*Δ*ldh* SD and *C. crenatum*Δ*ldh* SDA showed a higher AC and 2,3-BD selectivity, respectively, some pyruvate still converted to acetic and succinic acid. Therefore, increased AC and 2,3-BD production can be realized by disruption of the acetate and succinate biosynthesis pathway, as demonstrated in *K. oxytoca* and *B. subtilis* [11, 12]. Moreover, modification of key enzymes is critical for promoting cell metabolism. Previously, we have relieved the feedback inhibition of l-arginine by site-directed mutation of the key enzyme (NAGK) of *C. creantum*, and overexpressed the l-arginine operon, which effectively increased the yield of l-arginine by 41.7% [42] and 29% [43]. Therefore, the site-specific mutagenesis of butanol dehydrogenase (*butA*) gene might be further improved the AC and 2,3-BD yield and selectivity in *C. creantum*. In addition, although the repeated batch biocatalysis showed a high average productivity for AC and 2,3-BD production, the catalytic efficiency significantly deceased after only two batches, which can be further improved by cell immobilization to increase the cell viability and stability [44, 45]. On the other hand, process engineering, including buffer optimization, substrate concentration optimization, multiple biocatalysis strategies, and high cell density et al., can further improve AC and 2,3-BD production for commercial development. These synthetic and process engineering strategies can be applied to together to develop an efficient microbial cell factory for selective AC and 2,3-BD production in *C. creantum*.

## Conclusion

In this study, we successfully engineered *C. crenatum* SYPA5-5 to overexpress *alsS*, *alsD* and/or *bdhA* from *B. subtilis* 168 for selective AC and 2,3-BD biosynthesis from glucose. After depressing competition pathways, recombinant *C. crenatum*Δ*butA*Δ*ldh* SD and *C. crenatum*Δ*ldh* SDA further increased AC and 2,3-BD production to 76.93 g L^−1^ and 88.83 g L^−1^, respectively. Overall, respective non-natural AC and 2,3-BD biosynthesis pathway constructed in this study efficiently reduced the by-products accumulation and improved AC and 2,3-BD selectivity in *C. crenatum*. The optimal selection of AC and 2,3-BD biosynthesis strategies with further metabolic engineering should lead to the development of a promising microbial cell factory for AC and 2,3-BD production.

## Materials and methods

### Microorganisms and plasmids

All strains, plasmids and primers involved are shown in Additional file 6: Table S2 and Table 5, respectively. *B*. *subtilis* 168, *E. coli* BL21 and JM109, *C*. *crenatum* SYPA5-5 were stored in our laboratory. *E. coli* BL21 and JM109 were host strains of recombinant plasmid pXMJ19 and pk18, respectively. pXMJ19 is the shuttle expression vector of *E. coli* and *Corynebacterium*. pK18mobsacB carrying *sacB* gene is used for gene integration and knockout in *C. crenatum* [46].

**Table 5:** primers used in this study

Primer	Sequence (5′–3′) and restriction site
P*alsS*F	ACCG**GTCGAC**AAAGGAGGGAAATCATGACAAAAGCAACAAAAG (*Sal*I)
P*alsS*R	ACCG**GGATCC**CTAGAGAGCTTTCGTTTTC (*Bam*HI)
P*alsD*F	ACCG**GGATCC**AAAGGAGGGAAATCATGAAACGAgAAAGCAAC (*Bam*HI)
P*alsD*R	ACCG**GAATTC**TTATTCAGGGCTTCCTTC (*Eco*RI)
P*bdhA*F	ACCG**AAGCTT**AAAGGAGGGAAATCATGAAGGCAGCAAGATGG (*Hin*dIII)
P*bdhA*R	ACCG**GGATCC**TTAGTTAGGTCTAACAAGG (*Bam*HI)
P*alsSD*F	ACCG**CCCGGG**AA AGGAGGGAAATCATGACAAAAGCAACAAAAG (*Sma*I)
P*alsSD*R	ACCG**GAGCTC**TTATTCAGGGCTTCCTTC (*Sac*I)
P*butA*1F	ACCG**GAATTC**ATGAGCAAAGTTGCAATGGT (*Eco*RI)
P*butA*2R	ACCG**AAGCTT**CTAGTTGTAGAGCATGCCGC (*Hin*dIII)
P*butA*3R	TAGGCATTGACGGTGTGACCCTTGGCATCAATTGCACTGTCGAAATTAGC
P*butA*4F	GCTAATTTCGACAGTGCAATTGATGCCAAGGGTCACACCGTCAATGCCTA
P*ldh*1F	ACCG**GAATTC**ATGAAAGAAACCGTCGGT (*Eco*RI)
P*ldh*2R	ACCG**AAGCTT**TTAGAAGAACTGCTTCTG (*Hin*dIII)
P*ldh*3R	GTAGGATTGCGCGGGTGATGCGAGCCTTCGCAGTCAGCGTAGGTTCCCTT
P*ldh*4F	AAGGGAACCTACGCTGACTGCGAAGGCTCGCATCACCCGCGCAATCCTAC

Restriction sites are underlined and highlighted in bold

### Chemicals, mediums and cultivation conditions

FastPure Gel DNA Extraction Mini Kit, FastPure Plasmid Mini Kit and FastPure Bacteria DNA Isolation Mini Kit were all purchased from Vazyme (Nanjing, China). Antibiotics, restriction endonucleases and other tool enzymes such as high-fidelity 2× ExTaq DNA polymerase all purchased from Shenggong Biological (Shanghai, China). All other reagents of analysis grade or higher quality were obtained from Wuxi Reagent Company.

Luria–Bertani (LB) medium was used to culture *E. coli*. LBG (LB + 0.5% glucose) medium was used for preincubation of *C. crenatum*. *C*. *crenatum* competent medium for electroporation: LB medium was supplemented with 3% glycine and 0.1% tween 80. Shake flask fermentation medium (g L^−1^): (NH4)_2_SO_4_ 40, yeast extract 8, KH_2_PO_4_ 1.5, KCl 1, MnSO_4_·H_2_O 0.02, FeSO_4_·7H_2_O 0.02, MgSO_4_·7H_2_O 0.5, CaCO_3_ 30. Seed medium of 5 L bioreactor (g L^−1^): yeast extract 20, MgSO_4_·7H_2_O 0.5, (NH_4_)_2_SO_4_ 20, KH_2_PO_4_ 1.5. Fermentation medium of 5 L bioreactor (g L^−1^): (NH_4_)_2_SO_4_ 20, yeast extract 20, KH_2_PO_4_ 1.5, MnSO_4_·H_2_O 0.02, FeSO_4_·7H_2_O 0.02, MgSO_4_·7H_2_O 0.5. Resting cell bioconversion medium (g L^−1^): K_2_HPO_4_·3H_2_O 0.5, KH_2_PO_4_ 0.5, MnSO_4_·H_2_O 4.2, MgSO_4_·7H_2_O 0.5, FeSO_4_·7H_2_O 6. *E. coli* was cultured at 37 °C, 180 r min^−1^. *C. crenatum* was cultured at 30 °C, 180 r min^−1^ or 250 r min^−1^.

### Construction of Δ*butA* and Δ*ldh* fusion fragments

The *butA* gene fragment was obtained by PCR using the *C. crenatum* SYPA5-5 genome as template and PbutA1F and PbutA2R as primers. The PCR product was connected to pMD18-T cloning vector and sent to Shenggong Biological (Shanghai) for sequencing. Firstly, PbutA1F, PbutA3R and PbutA2R, PbutA4F primers were used for the first round of PCR using the *C. crenatum* SYPA5-5 genome as template to obtain two PCR products. Secondly, the deleted gene fragment Δ*butA* was obtained by fusing the two PCR products in the overlap-extension PCR and PbutA1F and PbutA2R were used (Additional file 7: Figure S5). The method for obtaining the deleted gene fragment Δ*ldh* was the same as above (Additional file 8: Figure S6).

### Construction of recombinant *C*. *crenatum*Δ*butA*Δ*ldh*

The deletion gene fragments Δ*butA* and Δ*ldh* were ligated to the pK18mobsacB suicide plasmid, respectively. Recombinant plasmids pK18-Δ*butA* and pK18-Δ*ldh* were constructed in *E*. *coli* JM109. The plasmid pK18-Δ*butA* was electroporated into *C*. *crenatum* according to the reported method [47]. The construction method of the *ldh* gene deletion strain was the same as above. The gene-deleted strains *C*. *crenatum*Δ*butA* and *C*. *crenatum*Δ*ldh* were obtained, respectively. The constructed homologous integration plasmid pK18-Δ*ldh* was electroporated into *C. crenatum*Δ*butA*, and after two homologous recombination, *butA* and *ldh* double gene deletion type recombinant strain *C. crenatum*Δ*butA*Δ*ldh* were obtained.

### Crude enzyme extraction and related enzyme activity assay

Recombinant *C. crenatum* cells were harvested by centrifugation at 8000 r min^−1^, 4 °C. Then cells were washed using 50 mM Tris–HCl buffer (pH 7.0). The washed cells were suspended in 5 mL of Tris–HCl buffer and lysozyme was added to treat the cell wall for 3–4 h. Cell disruption was accomplished by sonication for 30 min under ice bath conditions. Cell debris was removed by centrifugation at 4 °C, 12,000 r min^−1^ for 30 min and the supernatant was harvested for measurement of intracellular enzyme activity. Protein concentration in supernatant was determined by Bradford method [48]. ALS/AHAS, ALDC [49, 50], AR/BDH [51] and LDH [52] enzyme activities were determined according to the reported enzyme activity assay.

### Fermentation and resting cell bioconversion

The bacterial solution was inoculated into 10 mL LBG medium at a ratio of 1% and incubated for about 12 h. 3 mL bacterial liquid was transferred to 30 mL fermentation medium and cultured for 24 h. The cells in the fermentation medium were harvested and suspended in resting cell bioconversion medium containing 100 g L^−1^ glucose for resting cell bioconversion. 30 g L^−1^ CaCO_3_ was added during transformation to neutralize the organic acids produced. When resting cell bioconversion was performed in 5 L bioreactor, 4 L recombinant *C. crenatum* cultures were collected and resuspended in 2 L resting cell bioconversion medium. Resting cell bioconversion was performed in three batches at 30 °C, 250 r min^−1^. The converted solution was kept at pH 7.0 by feeding 50% NH_3_·H_2_O automatically. After the end of each batch of transformation, cells were harvested by centrifugation and then washed 3 times using Tris–HCl buffer. Resting cell bioconversion was continued by resuspending the cells in 2 L resting cell bioconversion medium containing 100 g L^−1^ glucose.

### Parameter measurement and analysis

For biomass analysis, the absorbance of cell cultures were measured at 562 nm with distilled water as the blank control. The dry cell weight (DCW) can be converted by using the equation (1 OD_562_ = 0.375 g L^−1^ DCW). The concentration of glucose in conversion solution and fermentation broth was determined using SBA-40E biosensor analyzer. The concentration of AC and 2,3-BD in conversion solution and fermentation broth was measured using Agilent gas chromatography [53]. Amino acids [28] and organic acids [54] were analyzed following the descriptions of previous reports.

## Additional files

**Additional file 1: Figure S1.** SDS-PAGE analysis of ALS, ALDC, and BDH in recombinant *C. crenatum*. The following samples and markers are shown: M: Protein marker; Lane 1: whole cell protein of *C. crenatum* WT; Lane 2: whole cell protein of *C. crenatum* S; Lane 3: whole cell protein of *C. crenatum* D; Lane 4: whole cell protein of *C. crenatum* A; Lane 5: whole cell protein of *C. crenatum* SD; Lane 6: *C. crenatum* SDA.

**Additional file 2: Figure S2.** Identification of pK18-Δ*ldh* by enzyme digestion. The following samples and markers are shown: M_1_: λDNA/*Hin*dIII marker; M_2_: DL2000 marker; Lane 1: pK18-Δ*ldh* digested with *Eco*RI; Lane 2: pK18-Δ*ldh* digested with *Eco*RI and *Hin*dIII.

**Additional file 3: Figure S3.** Identification of pK18-Δ*butA* by enzyme digestion. The following samples and markers are shown: M_1_: λDNA/*Hin*dIII marker; M_2_: DL2000 marker; Lane 1: pK18-Δ*butA* digested with *Eco*RI; Lane 2: pK18-Δ*butA* digested with *Eco*RI and *Hin*dIII.

**Additional file 4: Figure S4.** A: PCR identification of *ldh* gene knockout strains and recovery strains. M: DL2000 marker. Lane 1: *ldh* gene recovery strain. Lane 2 and 3: *ldh* gene knockout strains. B: PCR identification of *butA* gene knockout strains and recovery strains. M: DL2000 marker. Lane 1 and 4: *butA* gene recovery strains. Lane 2 and 3: *butA* gene knockout strains. C: PCR identification of *ldh* gene and *butA* knockout strain. M: DL2000 marker. Lane 1: PCR amplification of Δ*butA*. Lane 2: PCR amplification of Δ*ldh*.

**Additional file 5: Table S1.** Dry cell weight of *C. crenatum*Δ*ldh*, *C. crenatum*Δ*butA* and *C. crenatum*Δ*butA*Δ*ldh*. Results are shown as the mean ± standard of three replicates.

**Additional file 6: Table S2.** Strains and plasmids used in this study. Kanamycin resistance is labeled as Km^R^, and chloramphenicol resistance is labeled as Cm^R^.

**Additional file 7: Figure S5.** PCR amplification of the knockout fragment Δ*butA*.

**Additional file 8: Figure S6.** PCR amplification of the knockout fragment Δ*ldh*.

## Data Availability

All data involved in this study, if not found in this article or additional material, may be obtained from the corresponding author.

## Abbreviations

AC: acetoin
2,3-BD: 2,3-butanediol
ALS/AHAS: *α*-acetolactate synthase
ALDC: *α*-acetolactate decarboxylase
AR/BDH: 2,3-butanediol dehydrogenase
DA: diacetyl
LDH: lactate dehydrogenase
DCW: dry cell weight
