# Supplementary material for: Synthetic engineering of Corynebacterium crenatum to selectively produce acetoin or 2,3-butanediol by one step bioconversion method
Source: Microb Cell Fact. 2019 Aug 6;18:128. doi: 10.1186/s12934-019-1183-0 (PMC6683508; doi:10.1186/s12934-019-1183-0)
Supplement: Supplementary file 1 — Additional file 1: Figure S1. SDS-PAGE analysis of ALS, ALDC, and BDH in recombinant C. crenatum. The following samples and markers are shown: M: Protein marker; Lane 1: whole cell protein of C. crenatum WT; Lane 2: whole cell protein of C. crenatum S; Lane 3: whole cell protein of C. crenatum D; Lane 4: whole cell protein of C. crenatum A; Lane 5: whole cell protein of C. crenatum SD; Lane 6: C. crenatum SDA. [file 12934_2019_1183_MOESM1_ESM.docx]

**Additional material**

**Synthetic engineering of *Corynebacterium crenatum* to selectively produce acetoin or 2,3-butanediol by one step bioconversion method**

Xian Zhang^a,b^, Rumeng Han^a^, Teng Bao^b^, Xiaojing Zhao^c^, Xiangfei Li^a^, Manchi Zhu^a^, Taowei Yang^a^, Meijuan Xu^a^, Minglong Shao^a^, Youxi Zhao^d^, Zhiming Rao^a,^*

^a^ The Key Laboratory of Industrial Biotechnology, Ministry of Education, School of Biotechnology, Jiangnan University, 1800 Lihu Road, Wuxi 214122, Jiangsu, China.

^b^ Department of Chemical and Biomolecular Engineering, The Ohio State University, Columbus, OH 43210, USA.

^c^ School of Life Science and Technology ShanghaiTech University 393 Middle Huaxia Road Shanghai, China.

^d^ Beijing Key Laboratory of Biomass Waste Resource Utilization, College of Biochemical Engineering, Beijing Union University，Beijing，10023，P.R.China.

*Corresponding author at: The Key Laboratory of Industrial Biotechnology of Ministry of Education, School of Biotechnology, Jiangnan University, 1800 Lihu Road, Wuxi, Jiangsu 214122, China.

Tel: + 86-510-85916881; fax: + 86-510-85918516.

Email address:

zxshengwu@126.com (X. Zhang^a,b^); [2649770110@qq.com (R. M. Han^a^)](mailto:402237770@qq.com(C.L.Tan));

bao.166@buckeyemail.osu.edu (T. Bao^b^); zhaoxiaojing1990@126.com (X. J. Zhao^c^);

[1766187455@qq.com (X. F. Li^a^)](mailto:284077761@qq.com(Z.J.Zhu)); [1214008374@qq.com (M. C. Zhu^a^)](mailto:284077761@qq.com(Z.J.Zhu));

[ytw1228@163.com (T. W. Yang^a^)](mailto:ytw1228@163.com(T.W.Yang)); [xumeijuan@jiangnan.edu.cn (M. J. Xu^a^)](mailto:xumeijuan@jiangnan.edu.cn(M.J.Xu));

mlshao@jiangnan.edu.cn (M. L. Shao^a^) zhaoyouxi@buu.edu.cn (Y. X. Zhao^d^)

raozhm@jiangnan.edu.cn (Z. M. Rao^a,^*)


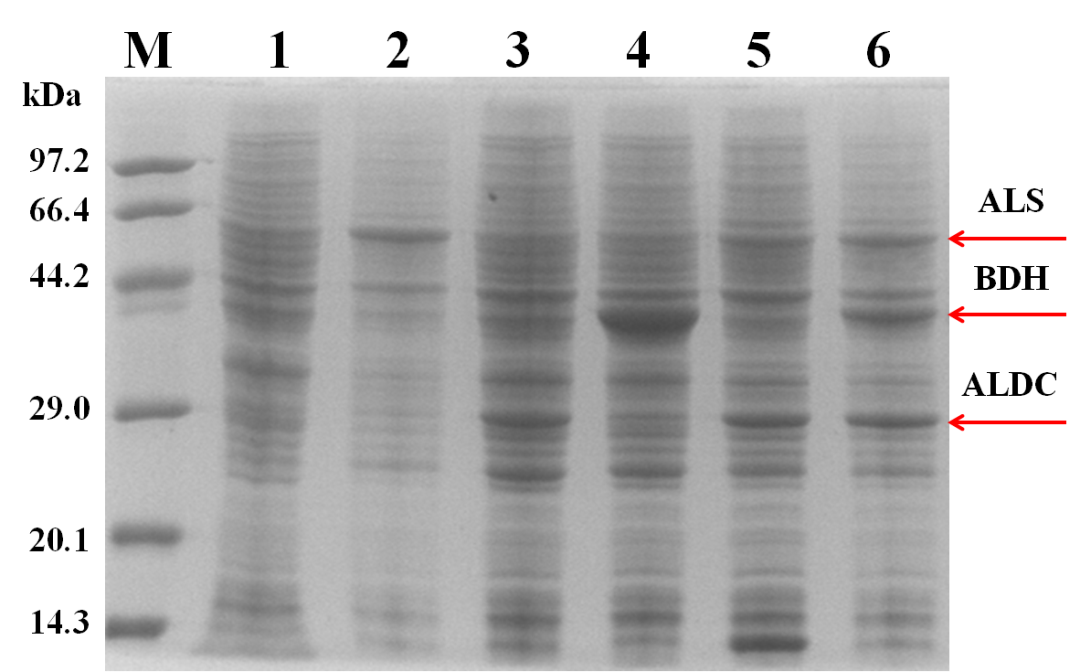


**Additional file 1: Figure S1 SDS-PAGE analysis of ALS, ALDC, and BDH in recombinant *C. crenatum*.** The following samples and markers are shown: M: Protein marker; Lane 1: whole cell protein of *C. crenatum* WT; Lane 2: whole cell protein of *C. crenatum* S; Lane 3: whole cell protein of *C. crenatum* D; Lane 4: whole cell protein of *C. crenatum* A; Lane 5: whole cell protein of *C. crenatum* SD; Lane 6: *C. crenatum* SDA.
