# Supplementary material for: Synthetic engineering of Corynebacterium crenatum to selectively produce acetoin or 2,3-butanediol by one step bioconversion method
Source: Microb Cell Fact. 2019 Aug 6;18:128. doi: 10.1186/s12934-019-1183-0 (PMC6683508; doi:10.1186/s12934-019-1183-0)
Supplement: Supplementary file 2 — Additional file 2: Figure S2. Identification of pK18-Δldh by enzyme digestion. The following samples and markers are shown: M1: λDNA/HindIII marker; M2: DL2000 marker; Lane 1: pK18-Δldh digested with EcoRI; Lane 2: pK18-Δldh digested with EcoRI and HindIII. [file 12934_2019_1183_MOESM2_ESM.docx]

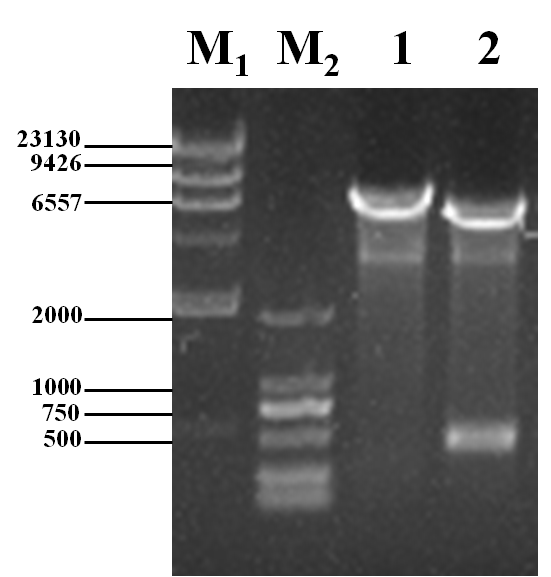


**Additional file 2: Figure S2 Identification of pK18-Δ*ldh* by enzyme digestion.** The following samples and markers are shown: M_1_: λDNA/*Hin*d III marker; M_2_: DL2000 marker; Lane 1: pK18-Δ*ldh* digested with *EcoR* I; Lane 2: pK18-Δ*ldh* digested with *Eco*R I and *Hin*d III.
