# Supplementary material for: Synthetic engineering of Corynebacterium crenatum to selectively produce acetoin or 2,3-butanediol by one step bioconversion method
Source: Microb Cell Fact. 2019 Aug 6;18:128. doi: 10.1186/s12934-019-1183-0 (PMC6683508; doi:10.1186/s12934-019-1183-0)
Supplement: Supplementary file 3 — Additional file 3: Figure S3. Identification of pK18-ΔbutA by enzyme digestion. The following samples and markers are shown: M1: λDNA/HindIII marker; M2: DL2000 marker; Lane 1: pK18-ΔbutA digested with EcoRI; Lane 2: pK18-ΔbutA digested with EcoRI and HindIII. [file 12934_2019_1183_MOESM3_ESM.docx]

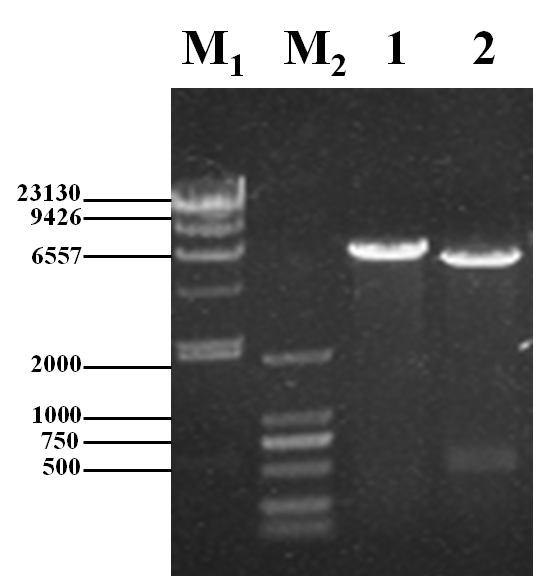


**Additional file 3: Figure S3 Identification of pK18-Δ*butA* by enzyme digestion.** The following samples and markers are shown: M_1_: λDNA/*Hin*d III marker; M_2_: DL2000 marker; Lane 1: pK18-Δ*butA* digested with *Eco*R I; Lane 2: pK18-Δ*butA* digested with *Eco*R I and *Hin*d III.
