# Supplementary material for: Synthetic engineering of Corynebacterium crenatum to selectively produce acetoin or 2,3-butanediol by one step bioconversion method
Source: Microb Cell Fact. 2019 Aug 6;18:128. doi: 10.1186/s12934-019-1183-0 (PMC6683508; doi:10.1186/s12934-019-1183-0)
Supplement: Supplementary file 4 — Additional file 4: Figure S4. A: PCR identification of ldh gene knockout strains and recovery strains. M: DL2000 marker. Lane 1: ldh gene recovery strain. Lane 2 and 3: ldh gene knockout strains. B: PCR identification of butA gene knockout strains and recovery strains. M: DL2000 marker. Lane 1 and 4: butA gene recovery strains. Lane 2 and 3: butA gene knockout strains. C: PCR identification of ldh gene and butA knockout strain. M: DL2000 marker. Lane 1: PCR amplification of ΔbutA. Lane 2: PCR amplification of Δldh. [file 12934_2019_1183_MOESM4_ESM.docx]

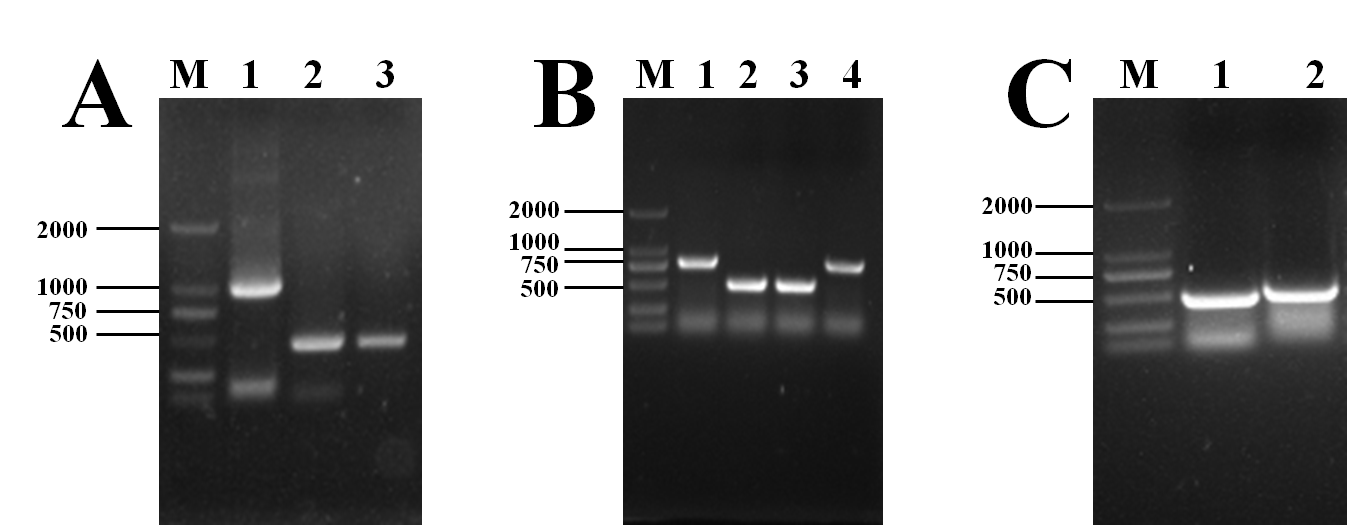


**Additional file 4: Figure S4 Figure S4-A: PCR identification of *ldh* gene knockout strains and recovery strains.** M: DL2000 marker. Lane 1: *ldh* gene recovery strain. Lane 2 and 3: *ldh* gene knockout strains. **Figure S4-B: PCR identification of *butA* gene knockout strains and recovery strains.** M: DL2000 marker. Lane 1 and 4: *butA* gene recovery strains. Lane 2 and 3: *butA* gene knockout strains. **Figure S4-C: PCR identification of *ldh* gene and *butA* knockout strain.** M: DL2000 marker. Lane 1: PCR amplification of Δ*butA*. Lane 2: PCR amplification of Δ*ldh*.
