# Supplementary material for: Synthetic engineering of Corynebacterium crenatum to selectively produce acetoin or 2,3-butanediol by one step bioconversion method
Source: Microb Cell Fact. 2019 Aug 6;18:128. doi: 10.1186/s12934-019-1183-0 (PMC6683508; doi:10.1186/s12934-019-1183-0)
Supplement: Supplementary file 5 — Additional file 5: Table S1. Dry cell weight of C. crenatumΔldh, C. crenatumΔbutA and C. crenatumΔbutAΔldh. Results are shown as the mean ± standard of three replicates. [file 12934_2019_1183_MOESM5_ESM.docx]

**Additional file 5: Table S1 Dry cell weight of *C. crenatum*Δ*ldh*, *C. crenatum*Δ*butA* and *C. crenatum*Δ*butA*Δ*ldh*.** Results are shown as the mean ± standard of three replicates.

| **Strain** | **Dry cell weight(g·L^-1^)** |
| --- | --- |
| *C. crenatum* WT | 10.78±0.17 |
| *C. crenatum*Δ*ldh* | 10.65±0.08 |
| *C. crenatum*Δ*butA* | 10.23±0.11 |
| *C. crenatum*Δ*butA*Δ*ldh* | 10.55±0.13 |
