# Supplementary material for: Synthetic engineering of Corynebacterium crenatum to selectively produce acetoin or 2,3-butanediol by one step bioconversion method
Source: Microb Cell Fact. 2019 Aug 6;18:128. doi: 10.1186/s12934-019-1183-0 (PMC6683508; doi:10.1186/s12934-019-1183-0)
Supplement: Supplementary file 6 — Additional file 6: Table S2. Strains and plasmids used in this study. Kanamycin resistance is labeled as KmR, and chloramphenicol resistance is labeled as CmR. [file 12934_2019_1183_MOESM6_ESM.docx]

**Additional file 6: Table S2 Strains and plasmids used in this study.**

| **Strains and Plasmids** | **Characteristics** | **Source** |
| --- | --- | --- |
| **Strains** |  |  |
| ***Escherichia coli*** |  |  |
| *E. coli* JM109 | recA1, endA1, gyrA96, thi-1, hsdR17, supE44, relA1, Δ(lac-proAB)/F’(traD36, proAB^+^, lacIq, lacZΔM15) | Our lab |
| *E. coli* BL21 | F–ompT gal dcm lon hsdSB(rB-mB-) λ(DE3(lacI lacUV5-T7 gene 1ind1 sam7 nin5)) | Our lab |
| *E. coli*/pXMJ19-*alsS* | Cm^R^ | This study |
| *E. coli*/pXMJ19-*alsD* | Cm^R^ | This study |
| *E. coli*/pXMJ19-*bdhA* | Cm^R^ | This study |
| *E. coli* /pXMJ19-*alsSD* | Cm^R^ | This study |
| *E. coli*/pXMJ19-*bdhA* -*alsSD* | Cm^R^ | This study |
| ***Corynebacterium crenatum*** |  |  |
| *C. crenatum* SYPA5-5 | L-Arg production strain, His^-^, Gram-positive bacteria | Our lab |
| *C. crenatum*Δ*butA* | *butA* deletion strain | This study |
| *C. crenatum*Δ*ldh* | *ldh* deletion strain | This study |
| *C. crenatum*Δ*butA*Δ*ldh* | *butA* and *ldh* double deletion strains | This study |
| *C. crenatum*/pXMJ19-*alsS* | Cm^R^ | This study |
| *C. crenatum*/pXMJ19-*alsD* | Cm^R^ | This study |
| *C. crenatum*/pXMJ19-*bdhA* | Cm^R^ | This study |
| *C. crenatum*/pXMJ19-*alsSD* | Cm^R^ | This study |
| *C.crenatum*/pXMJ19-*bdhA*-*alsSD* | Cm^R^ | This study |
| *C.crenatum*Δ*butA*Δ*ldh*/pXMJ19-*alsSD* | Cm^R^ | This study |
| *C.crenatum*Δ*ldh*/pXMJ19-*bdhA*-*alsSD* | Cm^R^ | This study |
| **Plasmids** |  |  |
| pXMJ19 | *Escherichia coli* and *Corynebacterium* shuttle expression plasmid, tac promoter, Cm^R^ | Our lab |
| pXMJ19-*alsS* | Recombinant expression plasmid containing *alsS* gene | This study |
| pXMJ19-*alsD* | Recombinant expression plasmid containing *alsD* gene | This study |
| pXMJ19-*bdhA* | Recombinant expression plasmid containing *bdhA* gene | This study |
| pXMJ19-*alsSD* | Recombinant expression plasmid containing *alsSD* gene | This study |
| pXMJ19-*bdhA* -*alsSD* | Recombinant expression plasmid containing *bdhA* and *alsSD* gene | This study |
| pK18*mobsac*B | Integrated vector carrying *sacB* gene for *Corynebacterium* gene integration and construction of gene deletion strain, Km^R^ | Our lab |
| pK18-Δ*butA* | *butA* deletion fragment Δ*butA* linked to pK18mobsacB constructed recombinant integration plasmid, Km^R^ | This study |
| pK18-Δ*ldh* | The *ldh* deletion fragment Δ*ldh* is a recombinant integrative plasmid constructed by ligating pK18mobsacB, Km^R^ | This study |

Note: Kanamycin resistance is labeled as Km^R^, and chloramphenicol resistance is labeled as Cm^R^.
