# Supplementary figures and images for: Synthetic engineering of Corynebacterium crenatum to selectively produce acetoin or 2,3-butanediol by one step bioconversion method
Source: Microb Cell Fact. 2019 Aug 6;18:128. doi: 10.1186/s12934-019-1183-0 (PMC6683508; doi:10.1186/s12934-019-1183-0)

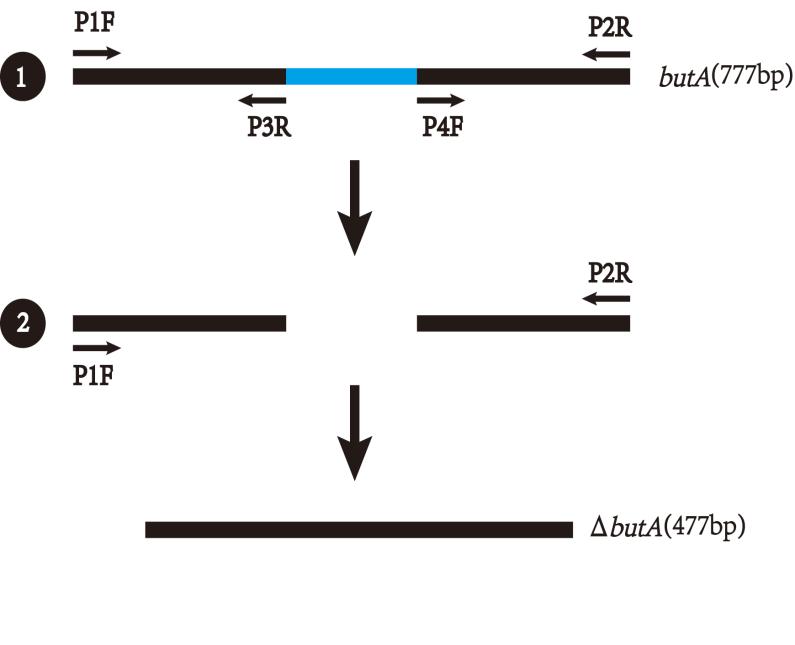


**Additional file 7: Figure S5 PCR amplification of the knockout fragment Δ*butA***

Supplement: Supplementary file 7 — Additional file 7: Figure S5. PCR amplification of the knockout fragment ΔbutA. [file 12934_2019_1183_MOESM7_ESM.docx]

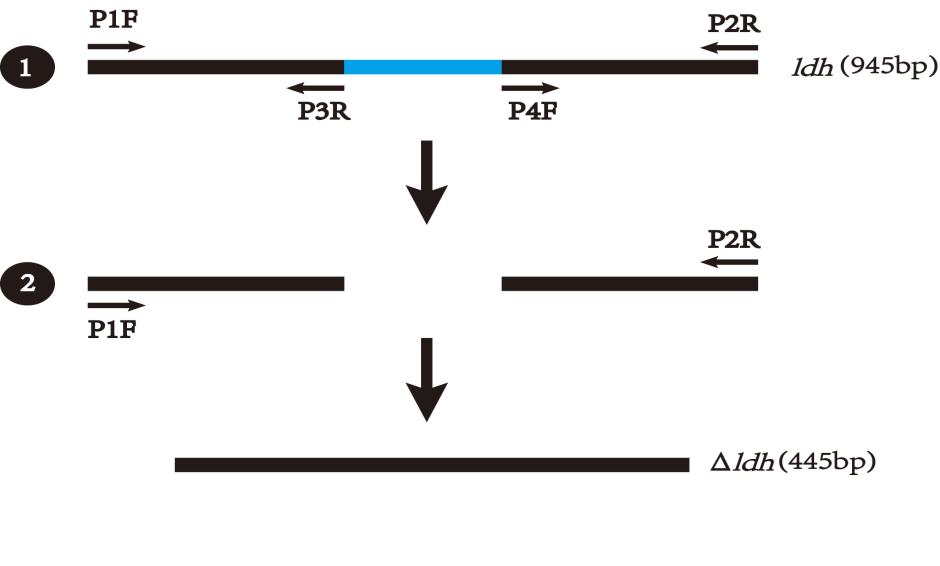


**Additional file 8: Figure S6 PCR amplification of the knockout fragment Δ*ldh***

Supplement: Supplementary file 8 — Additional file 8: Figure S6. PCR amplification of the knockout fragment Δldh. [file 12934_2019_1183_MOESM8_ESM.docx]
